# Overview of In-Hospital 3D Printing and Practical Applications in Hand Surgery

**DOI:** 10.1155/2021/4650245

**Published:** 2021-03-26

**Authors:** Marco Keller, Alissa Guebeli, Florian Thieringer, Philipp Honigmann

**Affiliations:** ^1^Hand Surgery, Department of Orthopaedic Surgery and Traumatology, Kantonsspital Baselland, 4410 Liestal, Switzerland; ^2^Medical Additive Manufacturing Research Group, Department of Biomedical Engineering, University of Basel, 4123 Allschwil, Switzerland; ^3^Department of Oral and Cranio-Maxillofacial Surgery, University Hospital Basel, Basel, Switzerland; ^4^Department of Biomedical Engineering and Physics, Amsterdam UMC, University of Amsterdam, Amsterdam, Netherlands

## Abstract

Three-dimensional (3D) printing is spreading in hand surgery. There is an increasing number of practical applications like the training of junior hand surgeons, patient education, preoperative planning, and 3D printing of customized casts, customized surgical guides, implants, and prostheses. Some high-quality studies highlight the value for surgeons, but there is still a lack of high-level evidence for improved clinical endpoints and hence actual impact on the patient's outcome. This article provides an overview over the latest applications of 3D printing in hand surgery and practical experience of implementing them into daily clinical routine.

## 1. Introduction

Three-dimensional (3D) printing, also known as Additive Manufacturing (AM), is a manufacturing technology which enables the production of three-dimensional models of a computer-designed template or data from medical imaging technologies by specially designed printers.

In 1981, a Japanese doctor, Hideo Kodama, developed a rapid prototyping technique, using a photosensitive resin that was polymerized by an UV light, creating the first 3D printing technique, an ancestor for SLA (stereolithography). In 1986, the first patent for SLA was submitted by Chuck Hull, and in 1988, two further 3D printing techniques were developed: SLS (“selective laser sintering”, in which powder grains are fused together locally by a laser) and FDM (“fused deposition modelling”, 3D printing with filaments) [1, 2].

In the following years, several additional methods were developed, including Binder Jetting and Polyjet, which are methods based on the inkjet printing technology, making color printing and combination of different materials possible [3].

3D printing was first used in the automobile, aerospace, and consumer product industries. Along with the radical improvements financed by these industries, new applications have been developed for its use in the medical field. According to the different aspects of every medical subspecialty, the implementation of 3D printing occurred with different pace and intensity. The applications of 3D printing in hand surgery are, compared to other subspecialties like for example craniomaxillofacial surgery, currently limited. In orthopaedic surgery, especially hand surgery, 3D printing enables the production of complex anatomical forms from data such as Computertomography (CT) images.

Current fields of application are the training of young hand surgeons, patient education, preoperative planning and fabrication of customized rehabilitation devices, customized surgical guiding tools, implants, and prostheses [4, 5].

An overview over the development and current implementations of 3D printing in each field is given in the subsequent paragraphs.

## 2. Printing Techniques and Materials

The entire 3D printing process consists of the following steps:

1. *Data Acquisition.* Acquisition of a 3D model of a medical image (CT, MRI, others) in DICOM format (Digital Imaging and Communications in Medicine)
2. *Image Processing.* Segmentation of the anatomy and transfer of DICOM into STL (stereolithography) format using a suitable software tool
3. *3D Slicing/Printing.* Slicing the STL model into several 2D slices and printing the 3D model by layering the slices on top of each other using a suitable printing technique
4. *Postprocessing.* Depending on the printing technique, the printed 3D model needs to be finalized by removing excess material, increasing the mechanical strength, removing the support structures, and improving the object's appearance

In the following table, the most common printing techniques and their characteristics are listed (Table 1).

Advantages of SLA printing are the accurate surface, and the relatively cheap price, weaknesses are the time-consuming removal of excess material and the limited size of the printed objects.

The SLS printing method allows the use of many different materials, including metals and does not rely on supporting structures. Furthermore, up to 100% density can be achieved. However, its surface is porous, the production time is longer due to the heating process, and the price for metal printing can be very high.

The strengths of FDM printing are the low price, fast production, and the possibility to print low density and, thus, light objects. On the other hand, this method relies on supporting structures needed to attach the printed object to the printing platform, which results in more time consumed for postprocessing to remove these supports.

The great advantages of the binder and material jetting techniques are the possibility to print different colors and materials in one object. BJ, unlike Polyjet, does not need any supporting structures, but the printed objects are less force resistant [3–5].

Of the mentioned printing methods, FDM is the most common today. The relatively simple mechanism and affordable materials make it the most accessible printing process for nonprofessionals.

## 3. Practical Applications in Hand Surgery

### 3.1. Training

The beneficial effect of 3D-printed models within orthopaedic education is well described. Some high-quality studies were able to highlight this effect especially with the involvement of bones with complex anatomical structures like the pelvis or the spine [6–9]. In the field of hand surgery, only very few articles on 3D printing technology in the education of junior surgeons have been published so far. Two reports from the same study group presented a synthetic wrist procedural simulator (Wristsim®, Biomodex™, Paris, France) based on 3D printing technology. They were able to highlight its potential use in training of volar plating in distal radius fractures and distal radius shortening osteotomy but also recognized its inferiority to cadaver specimen training [10, 11].

**Figure 1:**
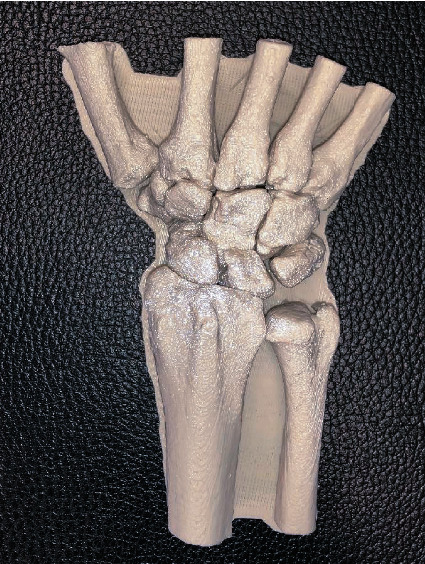
1 : 1 model based on 3D-Computertomography (CT) data of the carpal bones printed on an FDM-printer. This model allows analysis of intracarpal relationships und surgical training.

Despite the potential benefits, surgical training with models based on 3D printing technology has not found its way into hand surgery daily routine or even the curriculum of hand surgery specialisation yet. From our experience, we see the following reasons: only few clinics have the infrastructure and resources to provide every trainee with enough 3D-printed models and implants to ensure a good learning curve. Another reason is the complexity of the functional units: an isolated model of the distal radius might be useful to practice plating osteosynthesis or osteotomies. But due to the complex biomechanical units of the wrist and hand, hardly any other bone of the hand can be separated from its adjacent structures and still serve the purpose of a useful model to practice. The intercarpal relations are very complex and building an adequate model takes more effort than in the pelvis or the spine (Figure 1).

### 3.2. Patient Education

Comprehension of the injury is a cornerstone for a healthy patient-doctor-relationship. An adequate grasp of the extent of a patient's own lesion will help setting realistic expectations and increase the adherence to the proposed treatment. A tangible 1 : 1 model of, e.g., a fracture can facilitate the achievement of this goal. In a clinical trial on distal radius fractures, Chen et al. were able to show that not only were patients more likely to understand their condition and the operative plan, but the satisfaction and usefulness of the 3D prototype was even higher among patients than among surgeons themselves. This effect was measured with questionnaires and compared to the routine approach (patient education without the use of 3D-printed models) [12].

We find patient education with 3D-printed models especially useful in settings where patients suffered a complex, intra-articular fracture and need to understand how grave the damage to the joint is. In these cases, the printed model can simultaneously be used for preoperative planning.

### 3.3. Preoperative Planning

Numerous studies were published on the advantages of preoperative planning using 3D-printed models, and some were even able to highlight measurable improvements like shorter operation time, less intraoperative blood loss, and faster time to bony union [13, 14]. Preoperative planning can be roughly divided into visualizing and training with the help of three-dimensional models of a fracture or a soft tissue defect and the preoperative conduction of the actual surgery on a three-dimensional model including modifying (prebending or assembling) implants, which then are sterilized and used in the actual surgery resulting in shorter operation time.

In hand surgery research, the use of 3D-printed models has mainly been focussed on preoperative planning in patients with distal radius fractures or scaphoid pathologies. Bizzotto et al. reported that the preoperative planning (especially the placement of the fixation plate and screw orientation) with the use of 3D-printed models of distal radius fractures leads to substantial improvement in comprehension of the fracture. This effect was measured with a questionnaire to obtain feedback of the surgeon and was particularly observed in intra-articular fractures (with gaps or step of ≥2 mm or with a multifragmentary fracture pattern) [15].

With the conduction of a randomized controlled trial (RCT), Kong et al. were able to show that the use of a 3D-printed 1 : 1 model of a forearm with a distal radius fracture resulted in reduced operation time, intraoperative bleeding, and times of intraoperative fluoroscopy [16]. The exact same three beneficial effects could be shown in another RCT on “die-punch”-radius fractures [12].

Due to the complex three-dimensional shape of the scaphoid, the reduction and fixation of scaphoid fractures can be challenging. Jew et al. reported a series of four cases where preoperative planning with a 3D-printed model of the fracture facilitated the choice of approach, implant, and size of the cannulated screw [17]. Sometimes, despite adequate reduction and fixation, a scaphoid nonunion can occur. In recalcitrant scaphoid nonunions, the use of a vascularized osseous or osteocartilagineous graft from the medial femoral condyle (MFC) has proven to be a safe and reliable treatment option [18]. Some authors reported a method of proximal pole replacement with a vascularized osteocartilagineous MFC graft using a 3D-printed model of the graft based on CT-data from the contralateral uninjured hand. With this method, the harvesting and shaping of the graft can be conducted accurately and efficiently according to the authors [19, 20].

**Figure 2:**
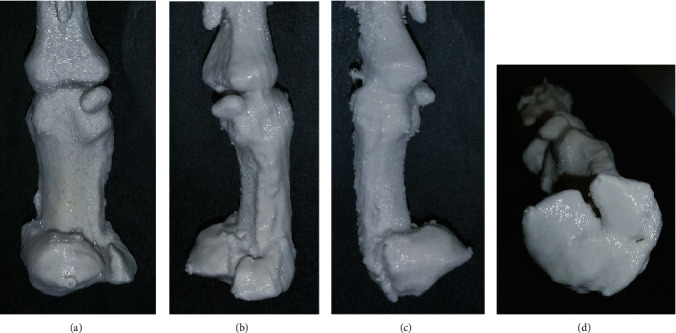
(a–d) 3D-printed 1 : 1 model of a displaced, multifragmentary, intra-articular fracture of the proximal part of the first metacarpal bone. The model was used to educate the patient about possible surgical treatment options and for preoperative planning.

**Figure 3:**
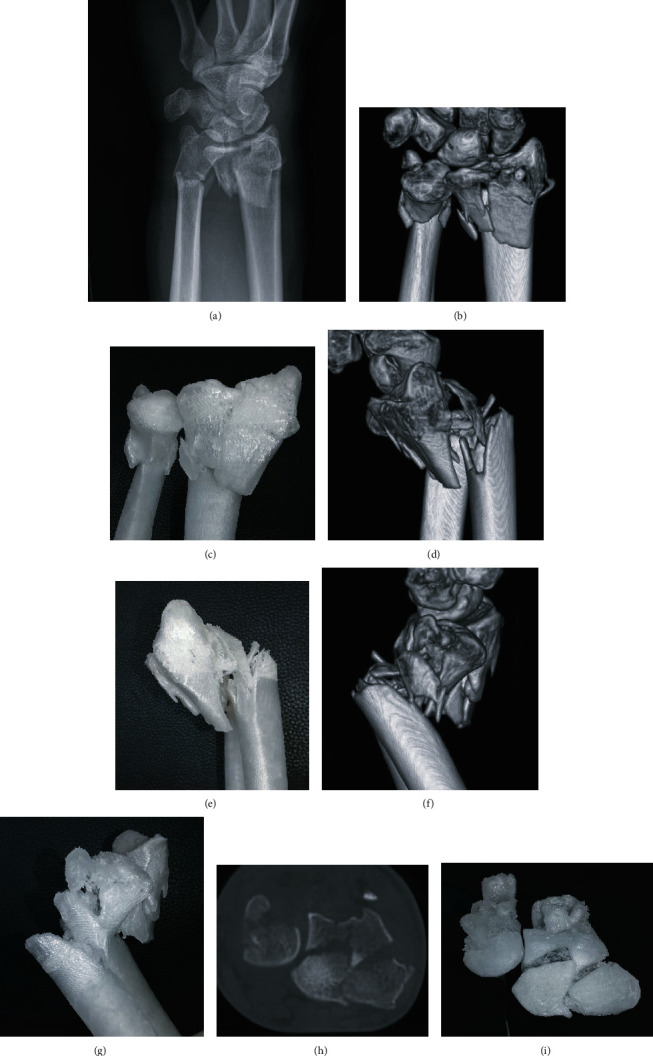
(a) Dorsopalmar radiographic view of a displaced, multifragmentary, intra-articular fracture of the distal radius, and ulna of a 45-year-old female. (b, c) Coronar view of the 3D-CT reconstruction and the equivalent view on the 3D-printed Polypropylene-model. (d, e) Sagittal view (radial aspect) of the 3D-CT reconstruction and the equivalent view on the 3D-printed Polypropylene-model. (f, g) Sagittal view (ulnar aspect) of the 3D-CT reconstruction and the equivalent view on the 3D-printed Polypropylene-model. (h, i) Axial CT-projection and the equivalent view on the 3D-printed Polypropylene-model allowing an overview on the intra-articular key fragments of the distal radius fracture.

In our institution, the planning of operations with 3D printing technology is mainly used for ORIF (open reduction with internal fixation) of dislocated intra-articular fractures of the radius or metacarpal fractures where the placement of single screws can be crucial (Figure 2). The models are printed with an FDM-printer based on 3D-CT images with high accuracy. A comparison of the dimensional accuracy of an isosymmetric-shaped test body printed with different technologies showed that FDM produces the highest precision (0.05 ± 0.005 mm) whereas SLS (0.11 ± 0.016 mm) and binder jetting (0.14 ± 0.02 mm) show a slightly lower but still satisfactory accuracy for surgical use [21]. A 1 : 1 model allows a more accurate assessment of the size and dislocation of key fragments than a sole analysis of CT-scans (Figure 3). The additional tactile and visual feedback provides valuable information on bony step-offs and gaps. However, this effect must not be overrated since much more factors than only fracture size and dislocation need to be taken into account. The relevance of single fragments varies according to attached ligaments, and the surgeon needs to be aware in which areas the reduction and fixation needs to be perfect (e.g., dorsal ulnar and volar ulnar corner in distal radius fractures) and in which areas minor gaps can be tolerated. This information cannot be provided by a 3D-printed fracture model. Furthermore, we do not see a major benefit of conducting the actual procedures on 3D-printed models and then sterilizing the implants, because most of the currently used implants in hand surgery fit very well and rarely need any bending or adaption which could be done prior to save time. This stands in contrast to other surgical subspecialties like for example craniomaxillofacial surgery where a retrospective survey showed that precontouring plates based on 3D-printed orbital models leads to a significant reduction of surgery time compared to intraoperative free-hand bending (57.3 ± 23.4 min vs. 99.8 ± 28.9 min, *p* = 0.001) in surgical repair of isolated orbital floor fractures [22]. With some experience, the choice of implant in hand surgery is mostly straightforward. So without having conducted an actual study on this distinct topic, we confirm the findings of Bizzotto et al. who reported no change in surgical decisions when 3D-printed models were used for the planning of ORIF in distal radius fractures [15]. There is also still a lack of studies that correlate presurgical planning using 3D-printed models with clinical endpoints to exhibit possible advantages for the patient compared to conventional planning.

### 3.4. Customized Braces/Splints

One of the most established applications of 3D printing technology in hand surgery is the fabrication of casts and splints. However, still no major widespread in hand surgery daily routine has taken place. Currently, most implementations of this technology happen within the framework of case series or feasibility studies. In our opinion, this is due to the following reasons: up to now, the use of digital design software is challenging and time-consuming; the printing process is lengthy and error-prone and hospitals without the possibility of in-house-printing rely on external suppliers which is costly. All in all, this effort mostly exceeds the effort of having a conventional plaster cast or splint built by far. A successful implementation of 3D-printed orthotics in daily routine requires the following premises: an intuitive and purpose-oriented designing software, a stable and fast printing process which is available around the clock, little required postprocessing but easy adaption if necessary, an efficient in-hospital workflow with collaboration of doctors and hand therapists and redundancy of skilled users at any level of the workflow, in case a person is unavailable (e.g., in the OR).

Most of the published papers on 3D-printed upper extremity orthotics focus on the composition of materials, the printing process, and feasibility. Up to date, no high-quality clinical trial was able to demonstrate the noninferiority or even superiority concerning wearing comfort or clinical outcome compared to conventional plaster casts and splints. Several in vitro [23, 24] and in vivo studies [25–29] highlight the safety and effectiveness of customized 3D-printed forearm casts. These trials indicate a good patient satisfaction due to light, breathable and waterproof splints. In another case series, Nam et al. highlighted the feasibility of 3D-printed finger splints for posthand burn patients [30].

**Figure 4:**
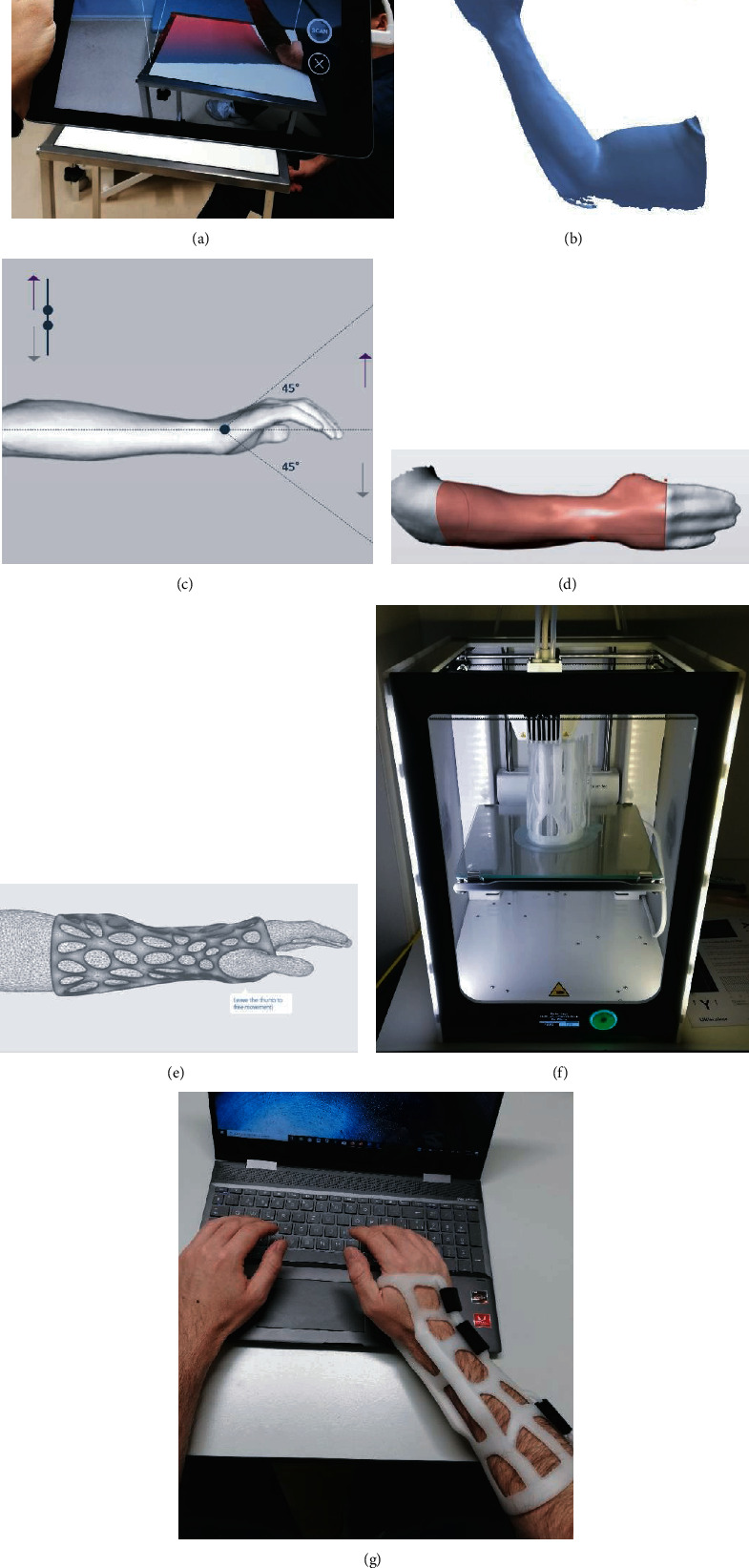
(a, b) 3D-scanning of the injured forearm using a tablet with an optical sensor (“Spentys© Point-of-Care Solution”, Spentys SA/NV, Brussel, BE). (c) Virtual adjustment of the wrist position if necessary (d, e) Designing of the forearm cast (“Polycast©” [Spentys SA/NV, Brussels, BE]) and generating an STL-file. (f) In-hospital, overnight printing using an FDM-printer with Polypropylene- (PP-) filament. (g) Fitted customized forearm-cast with ventilation openings and Velcro-Fasteners.

Our research group has implemented the use of 3D-printed hand and wrist rehabilitation devices in daily routine. Furthermore, we initiated the (up to our knowledge) first prospective randomized clinical trial assessing the relevance, feasibility, safety, and patient comfort of 3D-printed forearm casts compared to conventional plaster casts in the nonoperative treatment of distal radius fractures. The patients are scanned in the outpatient clinic with a handheld device. The data of the 3D-scan are processed and sliced using a software (“Spentys© Point-of-Care Solution” [Spentys SA/NV, Brussels, BE]) from a software company specializing in medical orthoses and immobilization devices. The forearm casts (“Polycast©” [Spentys SA/NV, Brussels, BE]) are then printed overnight and in-hospital using a 3D-printer with FDM-technology and Polypropylene (PP) filament (Figure 4). The postprocessing of the cast including the application of Velcro fasteners is carried out by the investigators before putting them on the patients. The control group is treated with conventional plaster casts for immobilization by a professional plasterer. During follow-up visits in our out-patient clinic, the patient's comfort is assessed at multiple times using two questionnaires specialized for this purpose. Additionally, several other clinical and radiological endpoints are measured. The first patient feedbacks show a good acceptance and patient comfort in the group with 3D-printed casts with a relatively low price of approximately 6 US$ per cast. To reduce printing and postprocessing time substantially, we recently started to additionally use a DLP-(Digital Light Processing) printer which results in a price of approximately 20 US$ per cast.

### 3.5. Surgical Guides

3D-printed surgical guides are mostly used for internal fixation of fractures or corrective osteotomy of malunions. These customized guides are usually either prebent/fitted on 3D-printed templates of the malunion and later sterilized [31] or 3D-printed themselves based on a digital model of the malunited bone [32]. The aim is to facilitate the osteotomy based on a preoperatively planned ideal osteotomy location and angle. The desired result is usually based on the contralateral healthy side.

Patient-specific surgical cutting guides have been described for malunions of the distal radius [33–35], for malunions of the diaphyseal area of both forearm bones (with custom-made fixation plates) [32], and for malunions of the scaphoid [36]. The senior author of this article presented a method using an acrylate Kirschner wire guide in combination with an acrylate wedge template for distal radius malunions which allow to harvest a precisely suitable iliac crest bone graft [37]. In a retrospective assessment of the early clinical outcome, 3D-planned and guided single-cut osteotomies of the forearm proved to be an accurate and reliable method [38].

Customized 3D-printed guides have also been used for osteosynthesis of scaphoid fractures. Yin et al. presented a method using a 3D-printed glove-like patient-specific guiding template to allow 1-shot percutaneous fixation [39]. De Wolf et al. presented another 3D-printed targeting device for scaphoid fractures and were able to show on cadavers that it provides similar accuracy while significantly reducing intraoperative radiation exposure and procedure time [40].

Most of these studies have a descriptive character and lack a control group. A systematic review on three-dimensional virtual planning of corrective osteotomies of distal radius malunions identified the following issues: no clinical study comparing the results of 3D-planning techniques with conventional planning methods could be identified. While the authors highlighted the benefit of 3D-planning, most studies used conventional two-dimensional (2D) radiographs to assess the radiological result of the procedures. This might lead to underestimation of residual deformities. Furthermore, a great heterogeneity of different radius malunions was seen. The authors concluded that no full comprehension of the added value of 3D-planning in distal radius malunion corrective osteotomy can be achieved without randomized controlled trials [41].

### 3.6. Personalized Implants/Solutions for Bone Defects

Besides orthopaedic aids like personalized splints, the trend for customization has also gained widespread use in the production of surgical implants. 3D printing technology is well established in the field of plastic and reconstructive surgery, where it is used to fabricate individualized synthetic and biologic implants, regenerative scaffolds, and cell-specific tissues and organs [42]. In craniofacial surgery, the use of patient-specific implants made from polymethylmethacrylate (PMMA) has proven to be cost-effective and applicable in daily clinical practice [43]. Yet again in hand surgery, up to now, there is only a hand full of case reports and feasibility studies on this topic. The printed implants are mostly based on scans of the contralateral healthy side.

In 2017, Kim et al. compared a 3D-printed volar locking distal radius plate fabricated by laser sintering of titanium alloy powder with two conventional volar locking plates. Biomechanical testing showed that the 3D-printed plate had a significantly higher strength than conventional plates, yet the implant was not customized to the bone [44].

**Figure 5:**
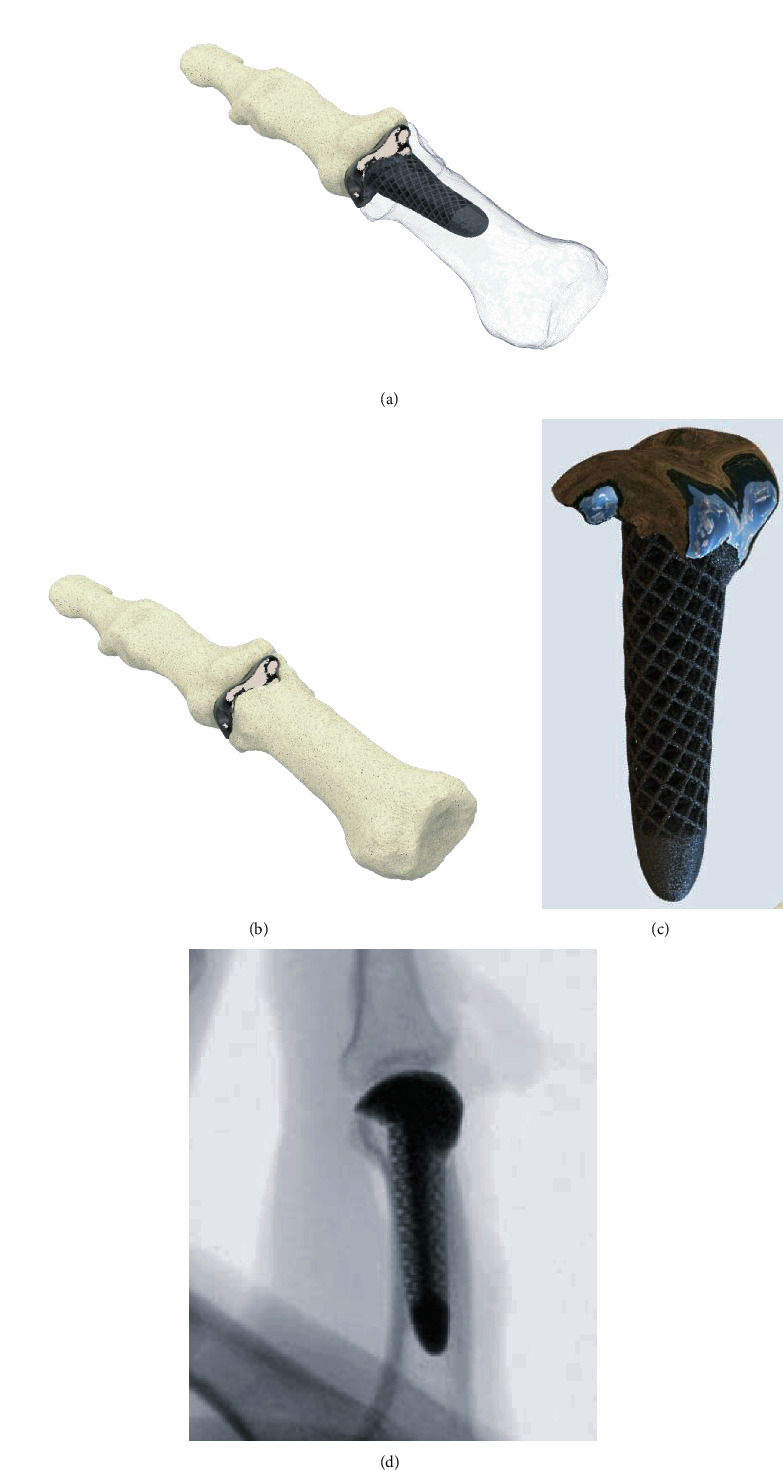
(a, b) Patient-specific 3D-printed partial joint replacement made of titanium (Xilloc Medical B.V., Sittard-Geleen, ND). (c) The shaft was designed with porous surface to enable osteointegration. (d) Lateral radiographic view of the finger after implantation of the patient-specific implant fitting precisely to the bone defect.

Other authors reported a 3D-printed (titanium) replacement of a finger proximal phalanx after recurrence of a giant cell tumor [45] or the replacement of the distal radius with customized, uncemented 3D-printed prostheses (Metal-PE (polyethylene)-combination with hydroxyapatite coating) in 11 patients with giant cell tumors [46]. Both report satisfactional functional outcomes with short-term oncologic salvage. Our own study group was able to replace parts of the proximal phalanx in a patient with a defect-lesion of the index finger after a chainsaw accident with a patient-specific implant. Since an “off the shelf” implant was no favourable option due to insufficient bone stock a patient-specific 3D-printed partial joint replacement made of titanium was used. The shaft was designed with a porous surface to allow osteointegration (Figure 5). The patient's range of motion in the proximal interphalangeal joint improved from preoperative 20° to postoperative 60°.

Carpal bones are complex in their anatomy and are at risk for fracture nonunion or avascular necrosis. Surgical treatment with nonvascularized or vascularized bone grafts is challenging. A prosthetic replacement of those bones could offer an appealing alternative. Xie et al. reported a case of patient-specific replacement of a collapsed lunate in stage IIIc Kienböck's disease. The implantation of a customized 3D-printed polyethylene spacer led to a nearly full range of motion and good pain relief 12 months after surgery [47]. The senior author of this article developed and showed the feasibility of a scaphoid prosthesis 3D-printed of titanium and ceramic and suggested 3D-printed Polyetheretherketon (PEEK) for further use [48, 49].

### 3.7. Personalized Prosthesis for Amputations

**Figure 6:**
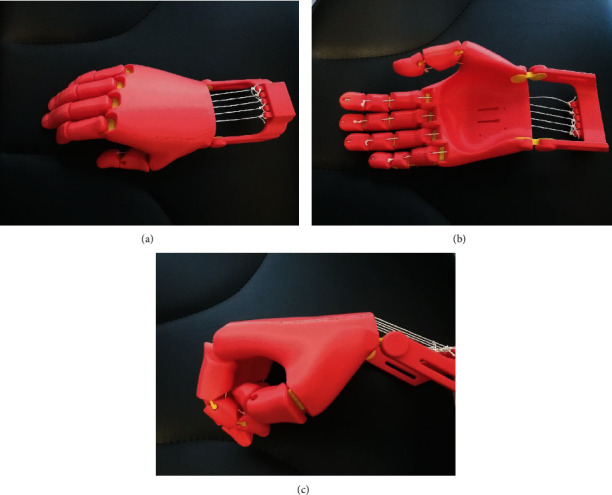
(a–c) “Flexy Hand 2”, a 3D-printed open source hand prosthesis (http://enablingthefuture.org/upper-limb-prosthetics/the-flexy-hand/).

(Partial) hand amputations are a unique entity and more common in developing countries. Providing patients with proper prosthetic replacement is often problematic in these areas. The feasibility of using 3D printing technology to fabricate hand prostheses was shown in several reports [50, 51]. Some authors laid their focus on providing children in developing countries and those with limited access to healthcare providers with reasonable prosthetic hand replacements [52]. Some of the designs were published online as open source files (Figure 6). Alturkistani et al. presented a 3D-printed prosthesis design with manufacturing costs of approximately 20 USD. Functional assessment showed that the prosthesis improved the patient's manual handling capabilities, especially regarding grasp stability [53].

## 4. Practical Issues

The eventual goal of implementing 3D printing technology in daily hospital routine is to enable mass production of customized splints, fracture models, and surgical guides. The workflow should be so efficient that it saves resources compared to conventional techniques. Until now, this goal is difficult to achieve in an in-hospital setting. According to our experience, problems can occur at every step of the process.

Even with intuitive and easy-to-use applications, the use of 3D printing technology requires extensive training for new users. Depending on the used printing technology, safety issues with flammable or potential harmful components need to be addressed. The workspace needs to be equipped with sufficient room, ventilation, stable temperature, and air humidity. Surface scanning devices need to be accessible and charged at any time. Frequently used printers need regular maintenance. Printers with repeated malfunctions need replacement. Software malfunctions or unexpected software updates can lead to delay which is particularly unfavourable if it leads to waiting time for a patient. Patients will show little acceptance for a new technology if it means more waiting time or additional hospital visits. The designing and slicing of 3D-models can either be done by the healthcare professional her/himself or an outside partner (e.g., an industrial partner specialized in 3D printing software). We recommend an interdisciplinary workflow where every medical and nonmedical specialist plays out his strength: the course of action is initiated by the surgeon with the request for a particular application, a radiologist should be responsible for acquisition and formatting of image data, a medical engineer should oversee the 3D-design process and printing and an occupational therapist should handle the fitting and postprocessing. With third parties involved, there are issues concerning data protection (location of the server storing patient data), financial relationship, and workflow efficiency (the fewer institutions involved the faster the process) that have to be settled.

Prior to the commercialization of a drug or medical device in the United States, FDA-clearance is required. To be referred as “FDA-Approved,” the manufacturer needs to prove substantial equivalence to a predicate device. This means that the performance and intended use of the new device is similar to a previously cleared device. To demonstrate effectiveness, often clinical data are needed which can be a considerable hurdle for manufacturers. In 2017, the FDA published the guidance document “Technical Considerations for Additive Manufactured Medical Devices” which provides information for manufacturers when working on a 3D-printed medical device regarding regulatory and quality assurance control [54, 55]. In Europe, the certification of medical devices needs to follow the European Union Medical Device Regulation (Council Regulation 2017/745 of 5 April 2017 concerning medical devices) which applies in all countries of the European union [56]. Additionally, these supraregional regulations also local regulations (e.g., Non-EU countries in Europe) need to be taken into account. We recommend not only the certification of the actual 3D-printed products but also certification of the manufacturing process through a third party. This will facilitate the expansion of applications and scaling of the project once effectiveness has been proven [57].

Postprocessing of splints or 3D-models requires specialized tools and can be very time-consuming. It needs to be planned carefully so it does not prolong the patient's outpatient clinic visit. Furthermore, it is important to plan a sufficient interval until the next visit of the patient to allow an additional printing attempt in case of failure of the first print. With FDM being the most common technology, this can mean an additional day.

3D-scans of tissue depict a snapshot at one certain moment of time. Increasing or decreasing soft tissue swelling, secondary dislocation of bones or other changes needs to be taken into account using customized splints (e.g., using PP that can be heated and adapted) or surgical guides based on skin or bone surface.

Another relevant potential problem is patient malcompliance. Since 3D-printed splints are mostly removable, there is an increased risk that the patient removes the splint deliberately during the immobilization period. This occurs less frequently using traditional circular plaster casts.

All of the abovementioned issues cause a significant amount of personnel and financial expenses which can initially be overwhelming compared to using conventional technologies. In our opinion, the efficiency can be maximized by conducting as many substeps as possible in-hospital, even if it requires substantial financial investments in the beginning. The workflow should be tested extensively and used on patients only when its stability is proved to guarantee good treatment quality.

## 5. Conclusion

Although the history of 3D printing technology is nearly 40 years old, its use is not yet well established in hand surgery compared to other medical subspecialities like craniomaxillofacial surgery or dentistry. Only in the last few years, interest among hand surgeons and the 3D printing industry has risen and intensive research has been initiated. Possible reasons for this delay are the following: in the eyes of the industry, hand surgery was not known to be a lucrative business investment. Hand surgeons relied on conventional proven products in their daily routine and did not see a significant potential benefit of the 3D printing technology for their work. With the general interest focussing more and more on patient-specific or personalized treatment, 3D printing became increasingly interesting for hand surgeons.

However, research on its use in hand surgery is still scarce. Up to now, complicated digital design software, lengthy and error-prone printing processes and expensive hardware were factors that inhibited a major widespread of 3D printing in daily routine. The idea that one person should be able to perform all substeps of the process, which require profound skills in different areas, might be another reason. For a successful implementation of 3D printing in daily routine, we therefore recommend the involvement of different medical and nonmedical specialists throughout the process. With today's complexity of digital design software programs, we found it to be most efficient to outsource the digital designing to closely collaborating medical engineers. At least with applications, where no complete automation is possible yet. In order to increase efficiency, postprocessing of printed objects can be handled by hand therapists, who often have more expertise in this area compared to hand surgeons.

The technical foundations for future applications such as bioprinting (replacement of tissue defects), in-hospital, or even in-OR implant-printing on demand are mostly known today. But due to missing clinical proof of effectiveness, governmental regulations, and too expensive and elaborate printing processes and materials their implementation in daily hand surgery routine is currently far from realistic. By simplifying workflows and reducing production costs, we believe that in the near future 3D printing technology can add a significant value to hand surgery.

## Figures and Tables

**Table 1:** Overview of different 3D printing techniques and their characteristics (SLA: stereolithography; SLS: selective laser sintering; DMLS: direct metal laser sintering; FDM: Fused deposition modelling; UV: Ultra-violet [3–5]).

Type of printing	Build material	Material distribution	Binding technique	Color printing	Material combination	Postprocessing			Material costs (US$ per kg, nonprofessional use)
						Remove excess	Increase strength	Remove support	
Photopolymerization (SLA)	Photopolymer (resin)	Vat	Light (laser)	No	No	Yes	Yes	Yes	50
Directed energy deposition (SLS, DMLS)	Powder	Bed	Heat (laser or welding)	No	No	Yes	No	No	45-75 (nonmetal) 350-550 (metal)
Material extrusion (FDM)	Filaments of plastic, food, paste, etc.	Extrusion	Heat (most often)	No	No	No	No	Yes	20-70
Binder jetting (BJ)	Powder	Bed	Jet adhesive	Yes	Yes	Yes	Yes	No	300-1000
Material jetting (Polyjet)	Photopolymer (resin)	Jet	Light (UV)	Yes	Yes	No	No	Yes	300-1000

## Data Availability

Readers can access any data underlying the findings of this research article by contacting the authors.
